# Comparative Study of Azithromycin Versus Doxycycline Effect on the Resistin Level in Periodontitis Patients With Type 2 Diabetes: A Randomized Controlled Clinical Trial

**DOI:** 10.7759/cureus.54849

**Published:** 2024-02-25

**Authors:** Jazia A Alblowi, Zienab S Farid, Mai S Attia

**Affiliations:** 1 Department of Periodontology, Faculty of Dentistry, King Abdulaziz University, Jeddah, SAU; 2 Department of Oral Medicine, Periodontology, Diagnosis and Radiology, Faculty of Dental Medicine, Al-Azhar University (Girls Branch), Cairo, EGY; 3 Department of Oral Medicine, Periodontology, Diagnosis and Radiology, Faculty of Dental Medicine, Misr International University, Cairo, EGY

**Keywords:** scaling and root planning, diabetes type 2, doxycycline, azithromycin, periodontitis

## Abstract

Aim: The present study aimed to determine if azithromycin (AZM) and doxycycline therapy, as an adjunct to scaling and root planning (SRP), modulate host response and improve clinical outcomes in periodontitis patients with type 2 diabetes mellitus (T2DM).

Patients and methods: Forty-five periodontal sites in 15 periodontitis patients with T2DM received nonsurgical periodontal therapy (NSPT). In Group I, patients were placebo (not receiving any medication), Group II patients received systemic AZM therapy (AZM 250 mg/day for five days), and Group III patients received doxycycline (20 mg twice per day for three months. The resistin level was collected and measured by enzyme-linked immunosorbent assay (ELISA). Gingival index (GI), probing depth (PD), and clinical attachment level (CAL) were recorded at baseline, one-month, and three-month intervals.

Results: All groups showed improvement in clinical parameters and resistin levels throughout the study. The mean resistin level at three months was the highest in Group I and the lowest in Group III. Patients in Group II showed a larger decrease in mean PD than those in Group I and III. Group III had the highest gain in mean CAL, with an increase of 1.78 mm in attachment.

Conclusion: Resistin might be a useful indicator of current disease status. In addition, benefits from adjunctive systemic use of AZM and doxycycline have been administered with non-surgical periodontal therapy.

## Introduction

Oral biofilm of different types triggers an immunoinflammatory response that leads to periodontal diseases. These diseases are marked by alveolar bone loss, attachment loss, and the downward movement of the junctional epithelium. The subgingival environment has a bacterial imbalance that causes periodontal disorders. Periodontal destruction results from the interaction of two main factors: the host-mediated immune-inflammatory response and the periodontally pathogenic biofilm [1].

Periodontal therapy is divided into four stages: systemic, initial, corrective, and maintenance [2]. Following the initial phase, the efficacy of mechanical debridement is evaluated in terms of locations with no inflammation, and a decision on the necessity for surgical intervention must be made [3,4]. Mechanical therapy is required for the disruption and elimination of dental biofilms, regardless of the methodology used. Other therapeutic techniques, such as host modulatory therapy, may, however, increase the benefits of mechanical therapy [5].

Periodontal disease is more common and severe in diabetics than in nondiabetics [6,7]. Moreover, glucose control in diabetics may be affected by periodontal infection [8], and glycemic control may improve after periodontal treatment [9], suggesting a two-way link between diabetes mellitus (DM) and periodontitis [10].

Some clinical trials have explored the use of antibiotics as an extra medication in nonsurgical periodontal therapy (NSPT) for diabetic patients with severe periodontal disease [10,11]. The effect of systemic antimicrobials in diabetic patients with periodontitis has been reviewed in two systematic reviews [12,13].

Doxycycline is an antibiotic that belongs to the tetracycline family and is effective against a wide range of bacteria, including those responsible for periodontal disease. It functions by inhibiting the production of proteins required for bacterial growth. When used in conjunction with dental cleaning, subantimicrobial doses of doxycycline (SDD) can help treat periodontal disease [14]. Subantimicrobial doxycycline can enhance the traditional subgingival debridement by strongly inhibiting the destruction of the extracellular matrix, even in cases of severe periodontitis. The Food and Drug Administration (FDA) has approved a dose of 20 mg twice a day for up to nine months for this type of doxycycline. There is no antimicrobial activity or antibiotic side effects (such as the emergence of antibiotic-resistant microorganisms) at the therapeutic levels that are acceptable [15].

The macrolide antibiotic azithromycin (AZM) is widely used in medicine to treat a variety of illnesses, including trachoma, middle ear, upper respiratory system, and sexually transmitted infections [16,17]. Additionally, it works well against the most typical periodonto-pathogens [16]. Because of their well-established immune-modulating/anti-inflammatory activities, macrolides are versatile beyond their antibacterial capabilities [18]. As a result, macrolides such as AZM are used to treat conditions including severe asthma, chronic obstructive lung disorders, and, more recently, cystic fibrosis that are not caused by bacteria [19]. Since neutrophils, macrophages, and particularly fibroblasts are key actors in the pathophysiology of the majority of periodontal disorders, AZM acquired its importance via its accumulation in these cells [20].

AZM may be a useful addition to non-surgical periodontal therapy for the treatment of periodontal diseases. This is due to its low plasma concentration, reduced gastrointestinal complications, effectiveness against common periodontal pathogens, simple dosing schedule, and potential interaction with host response mechanisms that help control the host’s response to periodontal pathogens [21]. Case studies have revealed remarkable clinical outcomes when AZM is used in conjunction with NSPT to treat advanced periodontitis [22].

Trial results that assess the benefits of AZM are still erratic. Numerous studies with durations of three to 12 months and dosages of 500-2,000 mg of AZM per day for three to seven days have revealed not only improvements in inflammatory, biochemical, and clinical parameters but also notable modifications in the make-up of subgingival biofilms, frequently leading to the eradication of Porphyromonas gingivalis [23-25]. A recent meta-analysis [26] also concluded that the benefit of AZM in reducing the probing depth (PD) of initially deep pockets can be observed after one year.

After being stimulated by the periodontal pathogen lipopolysaccharides, polymorphonuclear leukocytes, macrophages, and neutrophils are known to create the majority of resistin, an adipocytokine present in the inflammatory zone [26].

Resistin, an adipokine with a high cysteine content also known as adipocyte-specific secretory factor (i.e., ADSF), or found in inflammatory zone-3 (FIZZ3), was first identified in mouse adipose tissue [27,28]. It has a pro-inflammatory effect by increasing the release of TNF-alpha and IL-12, as well as inducing the nuclear translocation of the NF-kappa B transcription factor [29]. It increased the production of TNF-alpha, IL-6, and monocyte chemoattractant protein-1 (MCP-1) [30]. Periodontal disease is associated with higher serum levels of resistin [31]. Hiroshima et al. [32] found that chronic periodontitis (CP) patients had higher resistin levels in their serum than healthy people. Another study also showed that resistin levels in the gingival crevicular fluid (GCF) were higher in patients with CP than in the control [33]. Since resistin has been linked to insulin resistance and periodontitis, it may hold promise as an inflammatory mediator [30]. Hence, it is of great importance to record the resistin levels in gingival fluids from CP patients who have type 2 DM (T2DM).

The aim of this study was to compare the effects of systemic antibiotics as an adjunct to NSPT with NSPT alone on periodontal clinical parameters and resistin levels in diabetic patients with periodontitis, to see which one improves the outcome of NSPT.

## Materials and methods

A total of 45 periodontal sites were found in 15 individuals (nine females and six males), ranging in age from 25 to 51 years. Subjects were recruited through the Outpatient Clinics of the Department of Periodontology, Faculty of Dentistry, King Abdulaziz University, Saudi Arabia.

According to the American Academy of Periodontology standards, patients were clinically determined to have at least three locations with a clinical attachment level (CAL) of 5 mm and moderate-to-severe chronic periodontitis [34]. There were three groups in this study, with group under placebo (Group I) consisting of five patients with T2DM and periodontitis, Group II consisting of five patients with T2DM and periodontitis who received AZM dosage of 250 mg/day for five days after receiving an initial dose of 500 mg one hour before NSPT, and Group III consisting of five patients with T2DM and periodontitis who received doxycycline 20 mg twice daily for three months.

The study only included patients who had T2DM as the only systemic condition that could affect the periodontium or the periodontal treatment. The modified Cornell Medical Index [35] was used to determine the eligibility of the participants. They were not smokers, had not received any periodontal treatment, or had taken any antibiotics or anti-inflammatory drugs six months before the examination. Female patients were not pregnant or breastfeeding.

All subjects received information about the study's procedures and the benefits of their participation. All patients provided adequate written permission, indicating their agreement with the timetable study program design.

A single-blinded examiner assessed all periodontal clinical indicators at baseline and three months after NSPT. The gingival index (GI) was used to assess each patient's periodontal condition [27], PD [36], and CAL at the baseline, one-month, and three-month intervals.

Measurement of resistin levels

A human resistin enzyme-linked immunosorbent assay (ELISA) kit (Bio Vendor, Asheville, NC) was employed to measure the resistin level in GCF. The kit utilizes two polyclonal anti-human resistin antibodies: one of them is pre-coated on the microplate wells, and the other is biotin-labeled. After incubating and washing the standards and samples, the biotin-labeled antibody was added, followed by another incubation and wash. Next, streptavidin-HRP conjugate, substrate solution (TMB), and stop solution were prepended. Further, the absorbance of the yellow product was measured, and resistin concentration was calculated using the standard curve.

GCF samples were gathered from selected sites before, one month after, and three months after periodontal treatment. Samples were isolated and cleaned, and a sterile paper point was inserted into the gingival sulcus for 30 seconds. Following this, samples were transferred to Eppendorf tubes and stored at -80 °C. A human resistin kit and ELISA technique were employed to measure the resistin levels in the samples. Kits were utilized as per manufacturer instructions.

Non-surgical periodontal therapy

All patients underwent NSPT, which consisted of the following: supra and subgingival scaling and root planning that were done using an ultrasonic instrument. The periodontal treatment lasted for two weeks. The patients were instructed to brush their teeth twice a day and use interdental cleaning aids once a day for plaque control. The patients were also given chlorhexidine gluconate (0.12%)* as a mouthwash to use twice a day for two weeks. The patients were advised not to change their diabetic medications during the study period and to record any changes in their lifestyle, such as diet and exercise.

Human resistin quantification using ELISA

Resistin levels in human samples were quantified using a competitive ELISA kit (Sigma-Aldrich, Sofia, Bulgaria). The kit can analyze various samples, such as serum, plasma, and urine. The kit uses a microplate with an anti-rabbit secondary antibody, an anti-resistin antibody, a peptide standard (or target peptide), and a biotinylated resistin peptide. The standard and the sample peptides compete for binding to the resistin antibody and peptide on the microplate. The unbound biotinylated peptide binds to streptavidin-conjugated horseradish peroxidase (SA-HRP), which changes color. The color intensity is inversely related to the resistin peptide amount in the samples. A standard curve of known peptide concentrations was employed to estimate the peptide concentration in collected samples, and resistin concentration was calculated from the original data of the sample.

Statistical analysis

Clinical parameter values were provided as mean and standard deviation (SD). Because PD and CAL had normal (parametric) distributions, Student's t-test was used to compare the results of the studied groups. The paired t-test was used to examine the changes over time within each group. Since the GI and % change data had a non-parametric distribution, the Mann-Whitney U test was used. The Wilcoxon signed-rank test, a non-parametric alternative to the paired t-test, was employed to examine changes in resistin data over time within each group. One-way ANOVA was performed on results obtained in all groups. It is a method for comparing the means of the three groups. We can determine if the difference was significant or not using the p-value. Differences were set to be significant in case the p-value was less than 0.05.

## Results

Table 1 displays the variations in the GI measures for each group at baseline, one month, and three months following the NSPT. The maximum mean value of each GI was seen in all groups at baseline, and a decline was seen in all groups at various observation periods. Group III saw the most improvement, Group II experienced the second-highest improvement, and Group I experienced the least improvement, with a notable difference across the groups. Tables 2-3 show the results of ANOVA analysis and Tukey post-hoc results of the obtained data, respectively.

**Table 1:** Comparison of the gingival index (GI) in different groups (G1, G2, and G3) at baseline, one month, and three months of treatment. *Significant at p<0.05

		Mean	Sd. Dev.	Sd. Error	95% Confidence Interval for Mean		Min	Max	F-value	P-value
					Lower Bound	Upper Bound				
Baseline	G 1	2.29	0.47	0.13	2.02	2.56	2.00	3.00	0.421	*0.643
	G 2	2.22	0.43	0.1	2.01	2.43	2.00	3.00		
	G 3	2.5	0.53	0.17	2.12	2.88	2.00	3.00		
1 Month	G 1	1.43	0.51	0.14	1.13	1.73	1.00	2.00	0.456	*0.0756
	G 2	1.38	0.49	0.07	1.23	1.52	1.00	2.00		
	G 3	1.3	0.48	0.15	0.95	1.65	1.00	2.00		
3 Months	G 1	0.86	0.66	0.18	0.47	1.24	0.00	2.00	0.617	*0.0212
	G 2	0.56	0.7	0.17	0.21	0.91	0.00	2.00		
	G 3	0.10	0.41	0.1	0.13	0.33	0.00	1.00		

**Table 2:** One-way ANOVA of GI data in different treatment groups at different intervals.

Source	Sum of squares (SS)	Degrees of freedom (ν)	Mean square (MS)	F statistic	P-value
Treatment	104.8714	2	52.4357	16.7169	0.0035
Error	18.8201	6	3.1367		
Total	123.6914	8			

**Table 3:** Tukey HSD results of GI data in different treatment groups at different intervals. Here, A is data obtained at baseline, B is data obtained after one month, and C is data obtained after three months. *Significant at p<0.05; **Highly significant at p<0.01

Treatments pair	Tukey HSD Q statistic	Tukey HSD p-value	Tukey HSD inference
A vs B	5.3821	0.0207392	*p<0.05
A vs C	8.0226	0.0031034	**p<0.01
B vs C	2.6405	0.2275781	Insignificant

All groups showed an improvement in PD and CAL at one month and three months (p<0.05; Table 4). However, with regard to the greatest reduction in pocket depth measurement and clinical attachment level gain, there was a significant difference between groups at one month and three months, favoring doxycycline group (Group III) (p<0.05). Additionally, Table 4 illustrates that the mean pocket depth within Group Ⅲ revealed a statistically significant decrease from baseline (5.3 ± 0.48) to one month (4.3 ± 0.48) and three months of treatment (3.5 ± 0.70), respectively, with a total reduction in probing depth to about 1.8 mm.

**Table 4:** Comparison of pocket depth (mm) in different groups (G1, G2, and G3) at baseline, one month, and three months of treatment. *Significant at p<0.05; mm - millimeter

		Mean	Sd. Dev	Sd. Error	95% Confidence Interval for Mean		Min	Max	F-value	P-value
					Lower Bound	Upper Bound				
Baseline	G 1	5.21	1.1	0.3	4.58	5.85	3.00	6.50	4.38	*0.001
	G 2	4.56	1.22	0.29	3.95	5.16	3.00	6.50		
	G 3	5.3	0.48	0.15	5.22	5.86	4.00	6.00		
1 month	G 1	4.71	0.96	0.26	4.16	5.27	3.00	6.00	4.87	*0.019
	G 2	3.89	1.02	0.24	3.38	4.40	3.00	6.00		
	G 3	4.3	0.48	0.15	3.76	4.86	5.00	6.00		
3 months	G 1	4	0.78	0.21	3.55	4.45	3.00	5.00	5.67	*0.05
	G 2	3	0.97	0.23	2.52	3.48	2.00	5.00		
	G 3	3.5	0.70	0.22	2.93	3.88	6.00	7.00		

**Figure 1:**
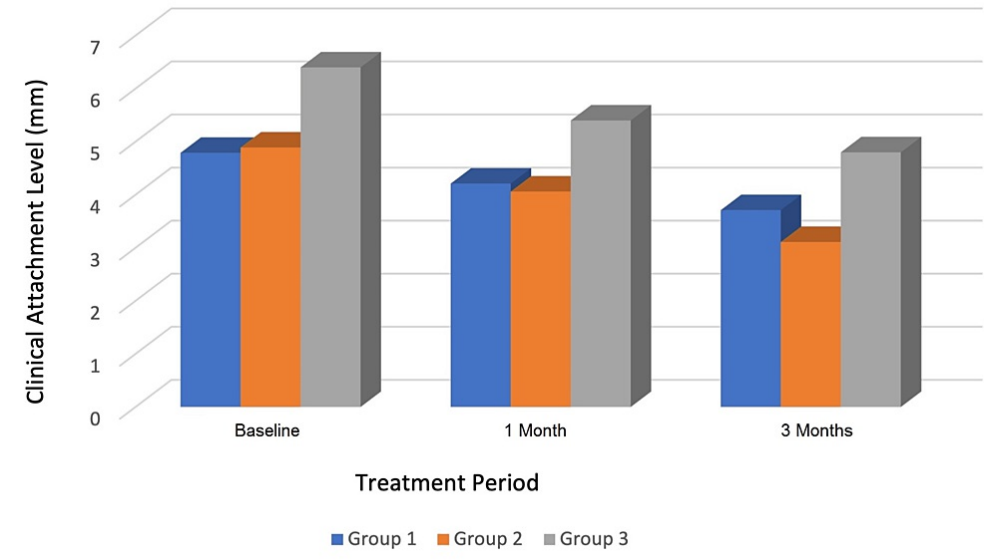
Comparison of the clinical attachment level in different groups at baseline and after one month and three months. mm - millimeter

Figure 1 represents the clinical attachment level in different groups throughout the study. Concerning the results of the mean clinical attachment level within Group Ⅲ, there was a statistically significant decrease at one month (5.4 ± 0.51) and three months (4.8 ± 0.78) when compared to the mean baseline value (6.4 ± 0.51), with a total clinical attachment gain of about 1.6 mm.

Changes in the GCF resistin level at one month and three months after periodontal treatment are shown in Table 5. At baseline, the mean resistin level was 19.68 ± 6.37 ng/mL in Group I and 22.38 ± 4.37 ng/mL and 18.7 ± 6.052 ng/mL in Groups II and III, respectively (p<0.05). The resistin level significantly decreased after periodontal therapy (p<0.05) in all groups.

**Table 5:** Resistin level (ng/mL) in different groups throughout the study. *Significant at p<0.05; ng/mL - nanograms per milliliter

		Mean	Sd. Dev.	Sd. Error	95% Confidence Interval for Mean		Min	Max	F-value	P-value
					Lower Bound	Upper Bound				
Baseline	G 1	19.68	6.37	1.7	16.01	23.36	11.18	29.34	0.413	*0.071
	G 2	22.38	4.37	1.03	20.21	24.55	15.26	30.01		
	G 3	18.7	6.052	1.43	0.043	0.104	0.45	0.189		
1 Month	G 1	15.4	5.34	1.43	12.32	18.49	8.03	23.29	0.153	*0.001
	G 2	15.65	3.23	0.76	14.04	17.26	11.94	20.20		
	G 3	13.2	4.57	1.075	0.033	0.042	0.030	0.044		
3 Months	G 1	14.05	4.71	1.26	11.33	16.77	8.44	22.61	0.854	*0.05
	G 2	12.03	2.58	0.61	10.74	13.31	8.03	15.98		
	G 3	10.07	0.16	0.04	0.013	0.022	0.008	0.029		

After periodontal treatment, an obvious significant decrease in the resistin level was recorded in all groups. In group I, the resistin levels were 15.4 ± 5.34 and 14.05 ± 4.71 at one month and three months, respectively. Group II showed a reduction in the resistin level from 15.65 ± 3.23 after one month to 12.03 ± 2.58 after three months. In the meantime, Group III showed the greatest reduction in the resistin level from 13.2 ± 4.57 after one month to 10.07 ± 0.16 after three months with a significant difference between the three groups postoperatively.

## Discussion

NSPT has been shown to be an effective treatment for reducing local inflammatory load [37]. The means of GI, PD, and CAL were considerably lowered following NSPT in this study. The findings agreed with earlier studies [38-40]. According to statistical analysis results shown in Tables 2-3, there was a significant difference between the GI data collected at baseline, one month, and three months of treatment. It is vital to remember that clinical indicators are related to an active diseased condition. Thus, it was hypothesized that individuals in all groups would respond to NSPT by improving clinical parameters. However, NSPT did not result in a greater reduction in mean CAL, which might be explained by the fact that CAL accumulation is more impacted by prior illness history than by the present periodontitis condition [41-43]. According to statistical analysis results shown in Tables 6-7, there was a significant difference between the PD data collected at baseline and three months of treatment. Moreover, in statistical analysis findings shown in Tables 8-9, there was a significant difference between the resistin level data collected at baseline and three months of treatment in different groups. Although NSPT improves clinical parameters, it may be inadequate, especially in particularly susceptible individuals, such as those with diabetes mellitus, to lower high levels of many underlying damaging inflammatory mediators. Thus, supplementary use of the host modulatory drug might improve clinical results in periodontitis therapy by downregulating several biological inflammatory mediators [44]. This study aimed to enhance the standard periodontal treatment by using host modulation therapy, which alters the harmful aspects of the immune inflammatory response of the host. This could lead to less periodontal damage and more periodontal stability, especially for people with systemic diseases such as diabetes. Host modulation therapy for periodontitis treatment involves taking SDD or AZM at a dose of 250 mg/day for five days after an initial dose of 500 mg one hour before NSPT.

**Table 6:** One-way ANOVA of PD data in different treatment groups at different intervals.

Source	Sum of squares (SS)	Degrees of freedom (ν)	Mean square (MS)	F statistic	P-value
Treatment	3.4838	2	1.7419	8.9921	0.0157
Error	1.1623	6	0.1937		
Total	4.6460	8			

**Table 7:** Tukey HSD results of PD data in different treatment groups at different intervals. Here, A is data obtained in Group I, B is data obtained in Group II, and C is data obtained in Group III at the baseline, one month, and three months of treatment. *Significant at p<0.05

Treatment pair	Tukey HSD Q statistic	Tukey HSD p-value	Tukey HSD inference
A vs B	2.8466	0.1894143	insignificant
A vs C	5.9948	0.0128236	* p<0.05
B vs C	3.1483	0.1443606	Insignificant

**Table 8:** One-way ANOVA of resistin level data in different treatment groups at different intervals.

Source	Sum of squares (SS)	Degrees of freedom (ν)	Mean square (MS)	F statistic	P-value
Treatment	104.8714	2	52.4357	16.7169	0.0035
Error	18.8201	6	3.1367		
Total	123.6914	8			

**Table 9:** Tukey HSD results of resistin level data in different treatment groups at different intervals. *Significant at p<0.05; **Highly significant at p<0.01

Treatment pair	Tukey HSD Q statistic	Tukey HSD p-value	Tukey HSD inference
A vs B	5.3821	0.0207392	* p<0.05
A vs C	8.0226	0.0031034	**p<0.01
B vs C	2.6405	0.2275781	Insignificant

Group III, which got SDD, exhibited the greatest improvement in all clinical measures. These findings might be explained by the fact that SDD can improve mechanical nonsurgical therapies by inhibiting metalloproteinases (MMPs), which are implicated in periodontal tissue destruction [45]. SDD also exhibits certain essential characteristics that may impede the evolution of periodontal disease, such as inhibition of MMPs [46-48], inhibition of the degranulation of polymorphonuclear (PMN) cells [47], and increased production of TGFβ1 [48].

AZM enhances the outcomes in Group II by penetrating more easily into inflamed tissues where fibroblasts and acute reactant cells such as neutrophils, macrophages, monocytes, and lymphocytes are present. AZM reduces the levels of proinflammatory cytokines, such as IL-1, IL-6, IL-8, and TNF-alpha, as well as growth factors, such as granulocyte-macrophage colony-stimulating factor. It also boosts the number of alveolar macrophages that are actively phagocytosing. The anti-inflammatory properties of AZM are due to the downregulation of these cytokines [17,49,50].

Numerous immune system functions are compromised by diabetes, which is also associated with sluggish immune responses and delayed recovery. Immune cell function changes brought on by diabetes lead to an inflammatory immune cell phenotype (suppression of macrophage growth factors and activation of pro-inflammatory cytokines from monocytes/polymorphonuclear leukocytes). As a result, there will be ongoing inflammation, more tissue disintegration, and less capacity for tissue repair [51]. Because resistin is a possible marker for inflammation and is influenced by inflammatory cytokines generated by periodontal pathogens in periodontitis, serum resistin levels in Group I were greater at one and three months compared to those in Groups II and III.

These findings were supported by the results of Tokuyama et al. [52] and Akram et al. [53] in 2017. The literature data revealed that patients with periodontitis and systemic inflammatory diseases such as diabetes had higher resistin levels than healthy people, but not higher than chronic periodontitis patients.

Following SRP, GCF levels of resistin significantly decreased over time in all groups, with the lowest mean value occurring at three months. It has been discovered that M1 macrophage infiltration produces inflammatory cytokines, including IL-6 and TNF-alpha that block insulin signaling [54]. Lean adipose tissue is linked to the predominant kind of anti-inflammatory M2 macrophages, which release arginase and IL-10 [55-57]. Furthermore, recent research has demonstrated that the transition from M2 to M1 macrophages is crucial for the development of islet dysfunction in T2DM [58]. Doxycycline administration has recently been found to minimize islet apoptosis, boost cell mitotic activity and percentage, and improve islet shape, particularly the cell-centric arrangement [59]. Furthermore, doxycycline therapy decreased islet inflammation as seen by fewer CD68 + macrophages, which reflect the M1 pro-inflammatory population. As a result, the greater improvement in resistin levels with SDD may be related to its anti-inflammatory impact.

The current study also found that Group II had much lower resistin levels in GCF than the control group. This could be because AZM changes the phenotype of macrophages, making them shift from M1 to M2 [60]. The M2 phenotype has a different activation pattern and helps to direct a Th2 humoral response and facilitate repair after inflammation. As mentioned before, resistin mainly comes from macrophages. AZM reduces the production of inflammatory cytokines by macrophages [61].

We have faced some limitations for this study, which are a small number of available publications that have the same interest in research and a small group of patients, including in our study, that make us recommend further investigations, research, and RCTs to make more evaluations.

## Conclusions

Based on the preceding findings, periodontitis patients with T2DM had greater levels of resistin in GCF. Non-surgical periodontal treatment reduced resistin levels in all groups. Furthermore, short-term usage of SDD or AZM was effective as an adjuvant to non-surgical periodontal treatment. Furthermore, combining NSPT with SDD led to greater reductions in GI, PD, CAL, and GCF resistin in periodontitis T2DM patients compared to periodontitis T2DM patients who did not get SDD and periodontitis T2DM patients who received AZM with NSPT.
